# Supplementary material for: Structural basis of SALM3 dimerization and synaptic adhesion complex formation with PTPσ
Source: Sci Rep. 2020 Jul 14;10:11557. doi: 10.1038/s41598-020-68502-4 (PMC7360590; doi:10.1038/s41598-020-68502-4)
Supplement: Supplementary file 1 — Supplementary information. [file 41598_2020_68502_MOESM1_ESM.docx]

**Supplementary Information**

**Structural basis of SALM3 dimerization and synaptic adhesion complex formation with PTPσ**

Sudeep Karki^1^, Alexander V. Shkumatov^2,3^, Sungwon Bae^4^, Hyeonho Kim^4^, Jaewon Ko^4^, Tommi Kajander^1^

^1^Institute of Biotechnology, University of Helsinki, Helsinki 00014, Finland

^2^Structural Biology Brussels, Vrije Universiteit Brussel, Brussels 1050, Belgium

^3^VIB-VUB Center for Structural Biology, Brussels 1050, Belgium

^4^Department of Brain and Cognitive Sciences, Daegu Gyeongbuk Institute of Science and Technology (DGIST), Daegu 42988, Korea

**
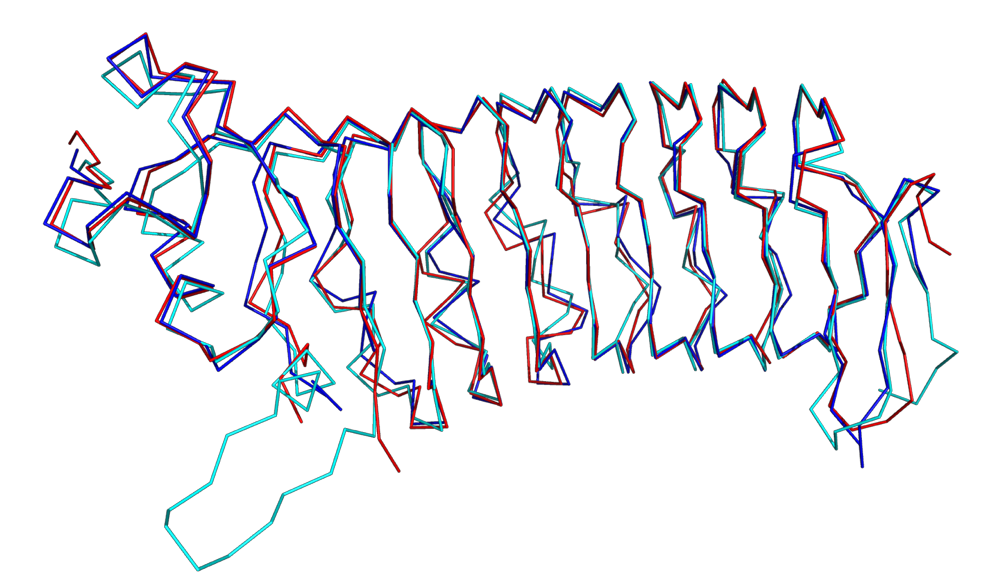
**

**Figure S1. Alignment of SALM LRR domains.** Alignment of SALM2 (PDB 5XWU) (red), SALM3 (blue) and SALM5 (PDB 5XNP) (cyan) LRR domains show that the structures are nearly identical. The SALM5 has the long loop connecting 7^th^ LRR repeat and LRRCT capping subdomain ordered in the crystal structure.

Figure prepared with PyMol, version 1.5.0.5 (www.pymol.org).


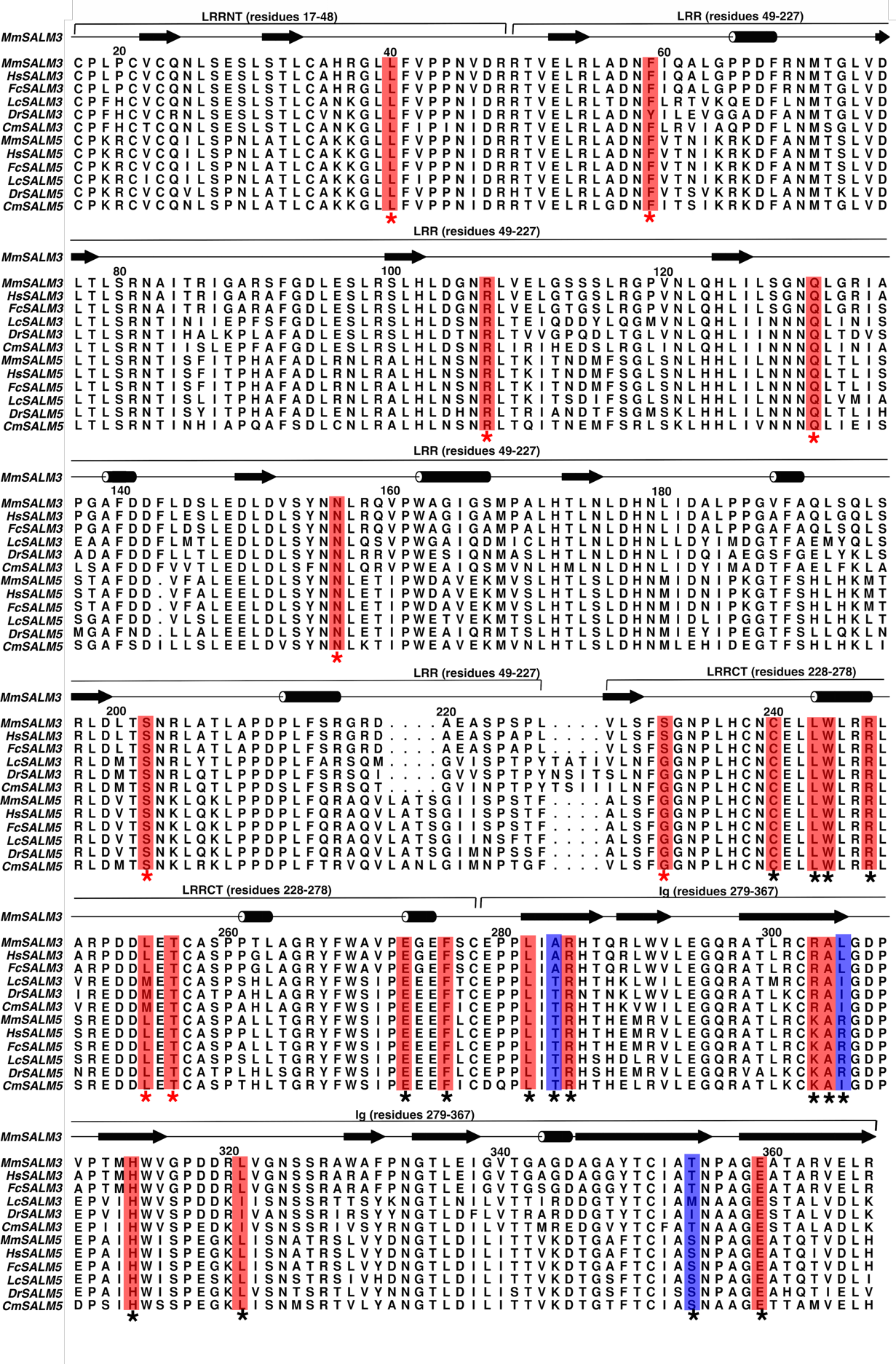


**Figure S2**. **Multiple sequence alignment of SALM3 and SALM5 from selected vertebrate species.** Alignment includes sequences from *Homo sapiens,* Human (Hs), *Felis catus,* cat (Fc), *Latimeria chalumnae*, coelacanth (Lc), *Danio rerio*, zebrafish (Dr) and *Callorhinchus milii*, shark (Cm). On top the secondary structure for the mouse SALM3 structure is shown. Amino acid sequence alignment consists of the SALM LRR and Ig domain regions. The residues located on the SALM3 LRR-dimer interface are labelled with red asterisks (*). The residues involved in SALM5:PTPδ interaction are labelled with black asterisks (*). Conserved residues in SALM3 and SALM5 present in the SALM3 LRR-dimer interface and SALM5-PTPδ interface are highlighted with red color and non-conserved residues with blue color.


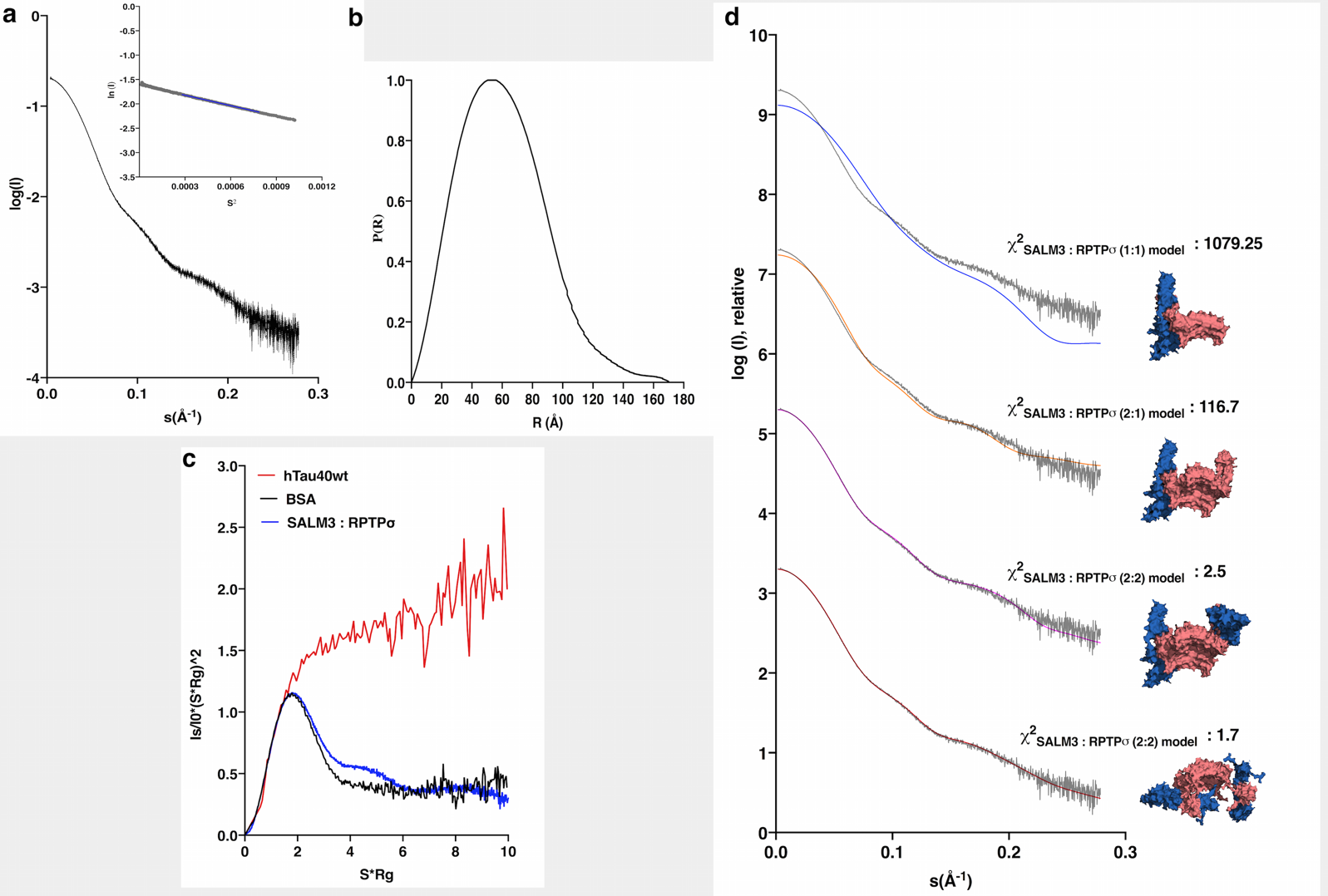


**Figure S3.** **SAXS analysis of the SALM3-RPTPσ complex.** A) Scattering profiles of SALM3-RPTPσ with the insert plot showing the Guiner region (trend line, blue color) with linear fits. B) Distance distribution function, P(r) of SALM3-RPTPσ complex. C) Dimensionless Kratky plots in comparison with globular BSA (grey) and natively unfolded human hTau40 protein (red). D) Scattering intensities profiles with corresponding fits to SALM3-RPTPσ complex models with different stoichiometry of SALM3 and RPTPσ. Surface structures show LRR domain (red) and RPTPσ (blue). χ^2^ values indicate the discrepancy between the experimental data and the scattering intensity calculated from the respective model. Experimental SAXS profiles were appropriately displaced along the logarithmic axis for better visualization and overlaid with corresponding fits. Molecular structure figures were prepared with PyMol, version 1.5.0.5 (www.pymol.org).


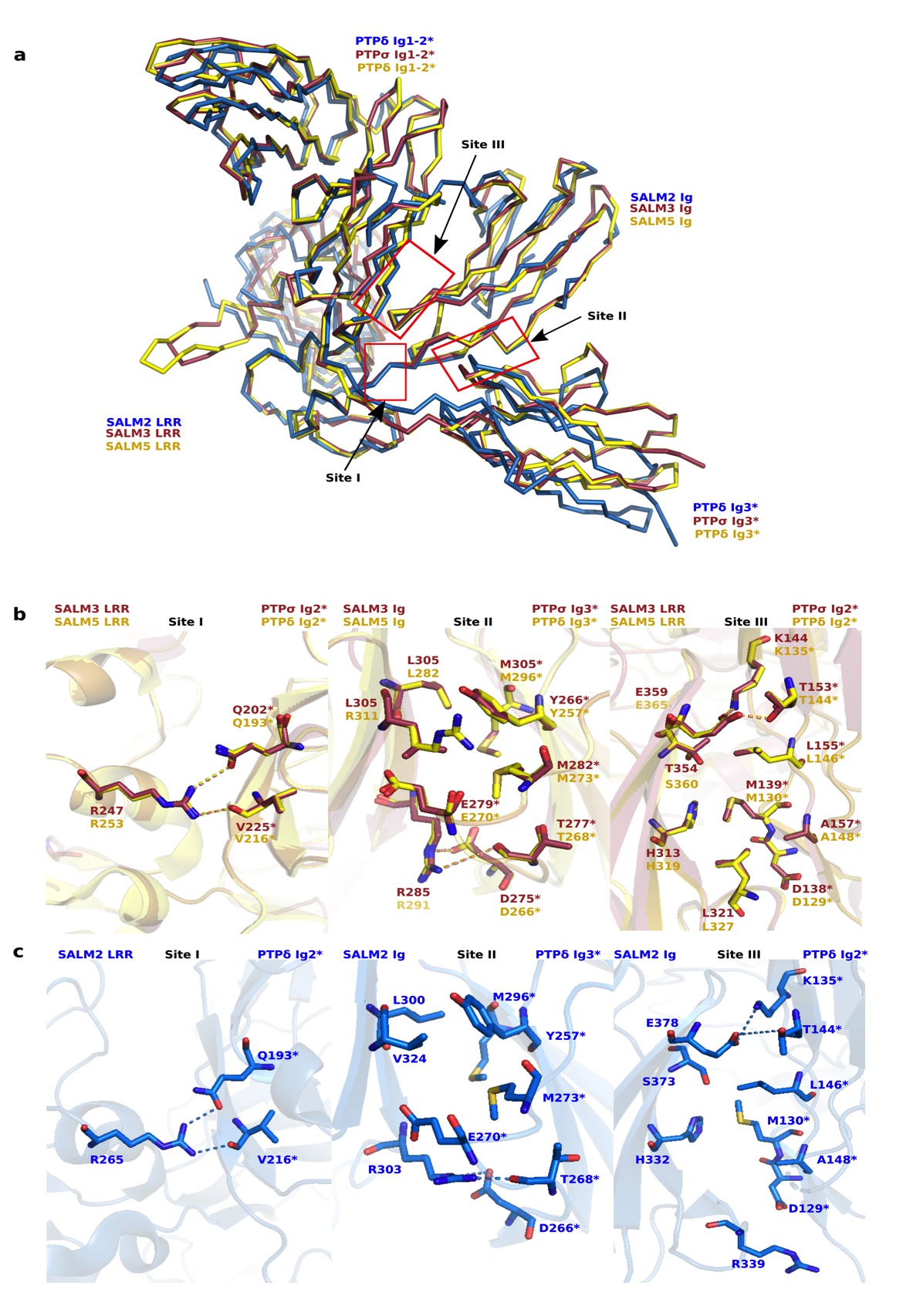


**Figure S4.** Alignment of the SALM2-PTPδ crystal structure (PDB ID: 5XWU) and SALM5-PTPδ crystal structure (PDB ID: 5XNP) to SALM3-PTPσ model. a) Structural alignment around the binding interface of the 1:1 complexes of SALM2-PTPδ (yellow) and SALM2-PTPδ (blue) to SALM3-PTPσ (red). The r.m.s.d.’s of the structural alignment of SALM3-PTPσ to SALM5-PTPδ, and SALM2-PTPδ to SALM5-PTPδ were 0.13 Å and 1.53 Å, respectively (due to SALM3 being modeled based on SALM5 crystal structure). Predicted interaction sites; Site I, Site II and Site III highlighted and labeled in Figure. b) Close-up view of the aligned interaction sites of the SALM3-PTPδ and SALM5-PTPδ complexes, from the left Site I, Site II and Site III as labelde. c) Interaction sites in the SALM2-PTPδ complex, as labeled in b). For visual clarity, non-carbon atoms are color-coded according to atoms (Oxygen, red; sulphur, yellow; nitrogen dark blue). "*" in the figure labels indicate residues in PTPδ or PTPσ in the complexes. Only residues Arg311 (SALM5), Arg339 (SALM2) identities differ significantly between the complexes. Molecular structure figures prepared with PyMol, version 1.5.0.5 (www.pymol.org).


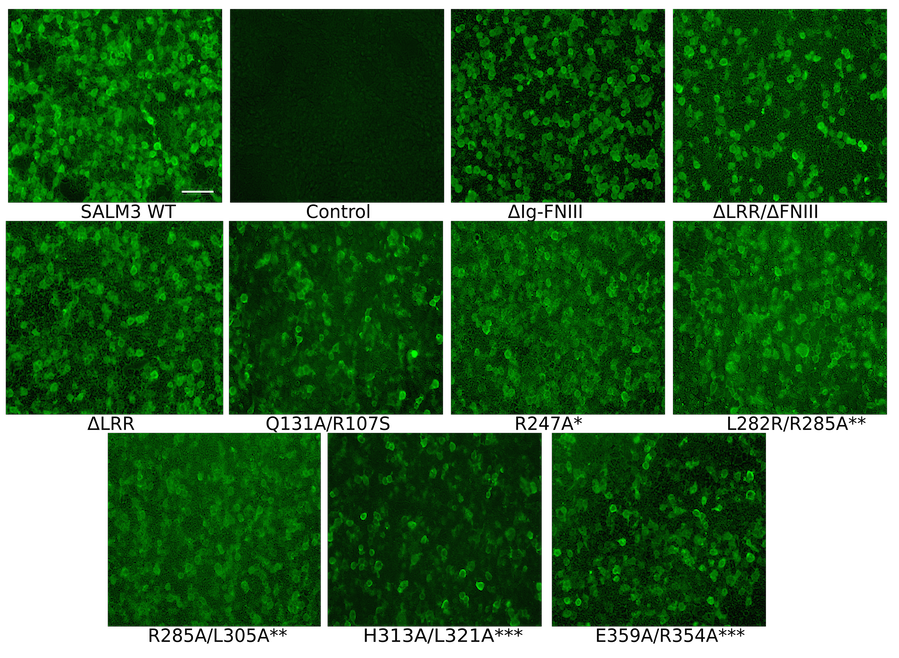


**Figure S5. Fluorescence microscopy imaging of the surface expression of SALM3 variants on the HEK293T cells for the cell binding assay**. Transfected cells were immunostained with rabbit anti-HA antibodies and detected with secondary antibody (Alexa 488 anti-rabbit, Molecular probes). Expression was detected with FloidTM Cell Imaging system. Scale bar: 125 μm (WT image; applies to all images). interaction site I is indicated with `*´ in the the SALM3-PTPσ,complex `**´ indicates site II, and `***´ indicates site III (as described in Figure 4).

**
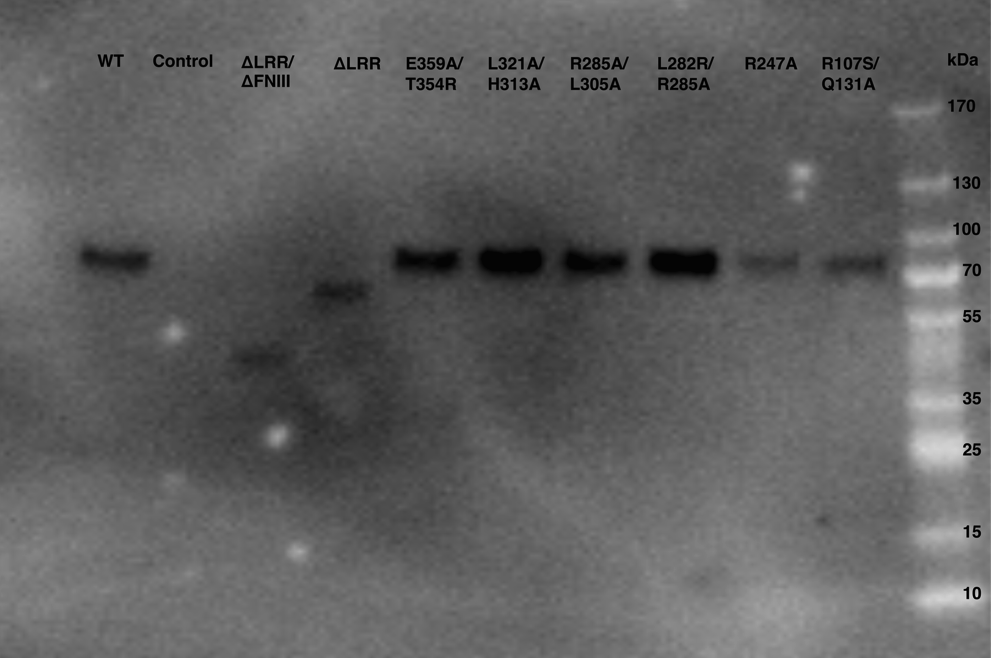
**

**Figure S6.** **Western blot analysis of secreted SALM3 variants in S2 *Drosophila* cells**. For western blot analysis, the SALM3 expressed cells were spin down, and 12 μl supernatant was loaded to the SDS-PAGE, expect for the deletion mutants, which were diluted first 1:10 due to initial much higher intensity. Supernatant from non-transfected cells was used as control (12 μl). Secreted proteins were detected with goat anti-human polyclonal HRP-conjugated antibody (Abcam ab98567).

**
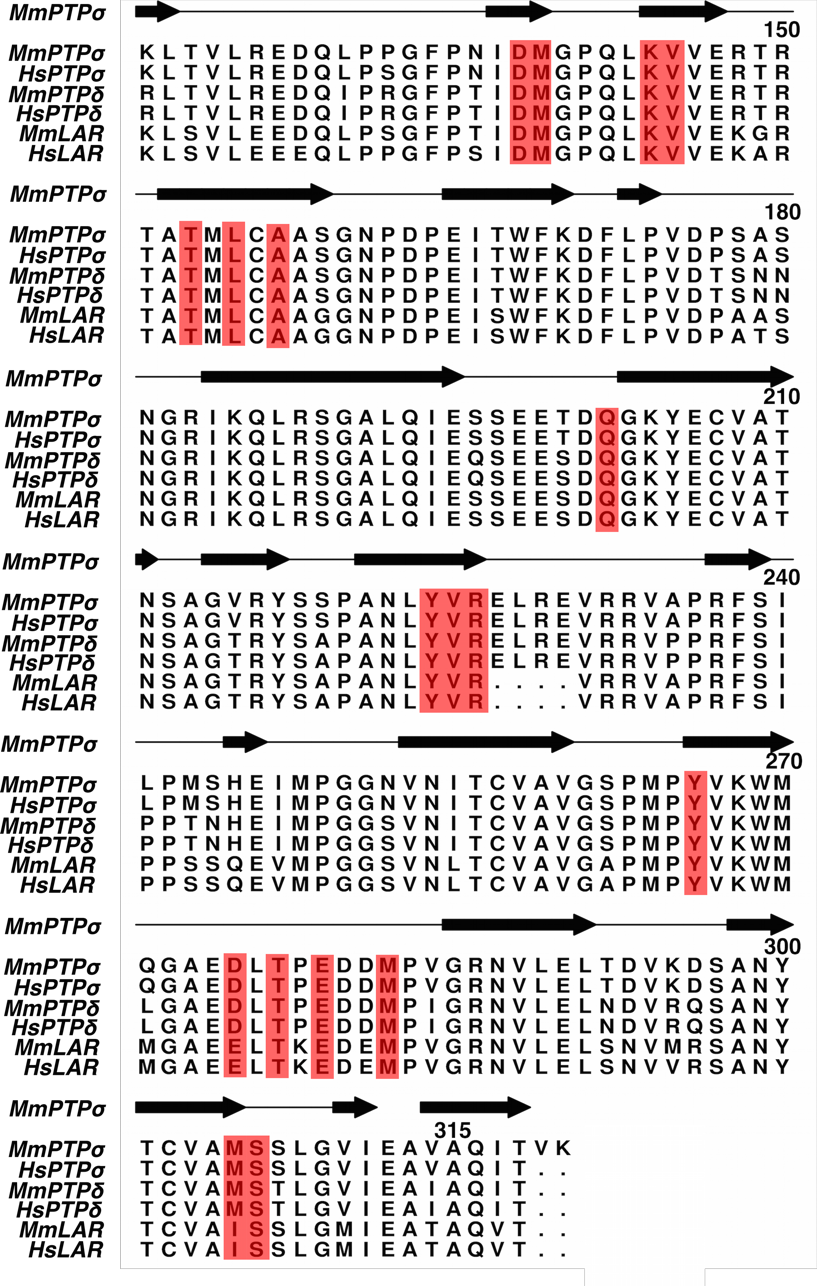
**

**Figure S7.** **Multiple sequence alignment of the three members of human and mouse LAR-RPTPs (LAR, PTPσ and PTPδ).** Alignment includes LAR-RPTPs Ig1-3 domains. PTPδ residues involved in SALM5 interaction and conserved in PTPσ are marked. Interface residues identified using PISA server with ≥ 75% buried area percentage. Color-coding as in Figure S2.

**
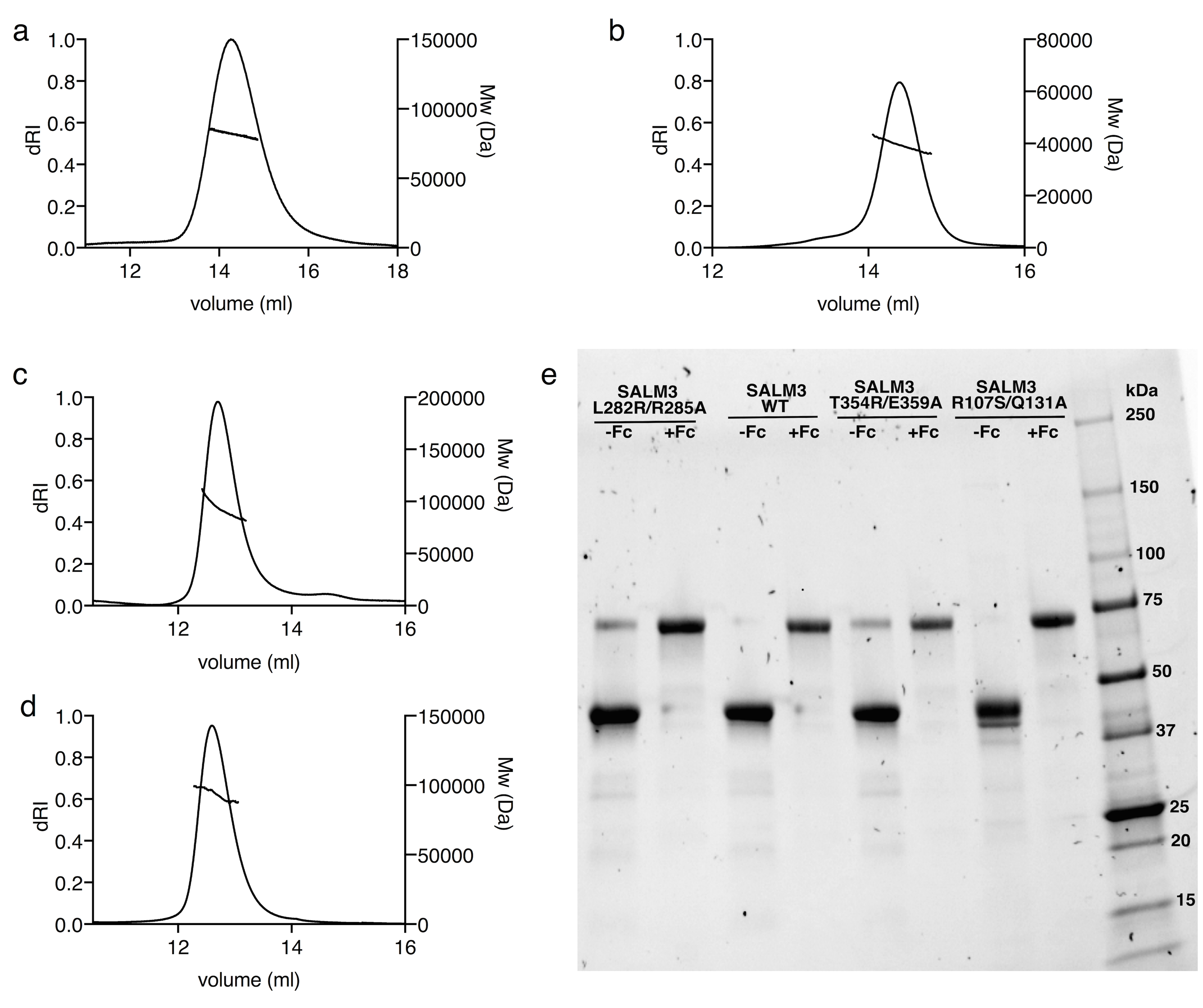
**

**Figure S8.** Characterization of oligomeric state of purified proteins of SALM3 LRR-Ig wild type and mutants by SEC-MALS. a) Wild type SALM3 LRR-Ig, b) SALM3 LRR-Ig R107S-Q131A constructs, c) SALM3 LRR-Ig L282R-R285A, d) SALM3 LRR-Ig T354R-E359A. Calculated Mws (right Y-axis) are plotted over the protein (dRI) signal peaks; average observed molecular weights were 39.1kDa for SALM3 LRR-Ig R107S-Q131A, 93.6 kDa for SALM3 LRR-Ig L282R-R285A, 95.2 kDa for SALM3 LRR-Ig T354R-E359A constructs, and 82.4 kDa for wild type SALM3 LRR-Ig. e) An SDS-PAGE analysis of SALM3 wild type LRR-Ig and mutant constructs in presence and after cleavage of the Fc-tag (as indicated in the figure).

**
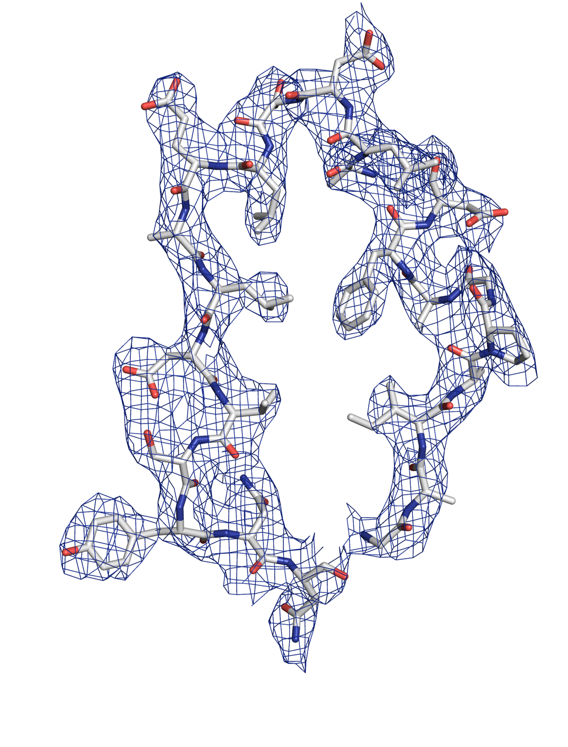
**

**Figure S9.** Representative 2F_o_-F_c_ electron density for the SALM3 LRR domain contoured at 1σ level. Figure prepared with PyMol, version 1.5.0.5 (www.pymol.org).

**
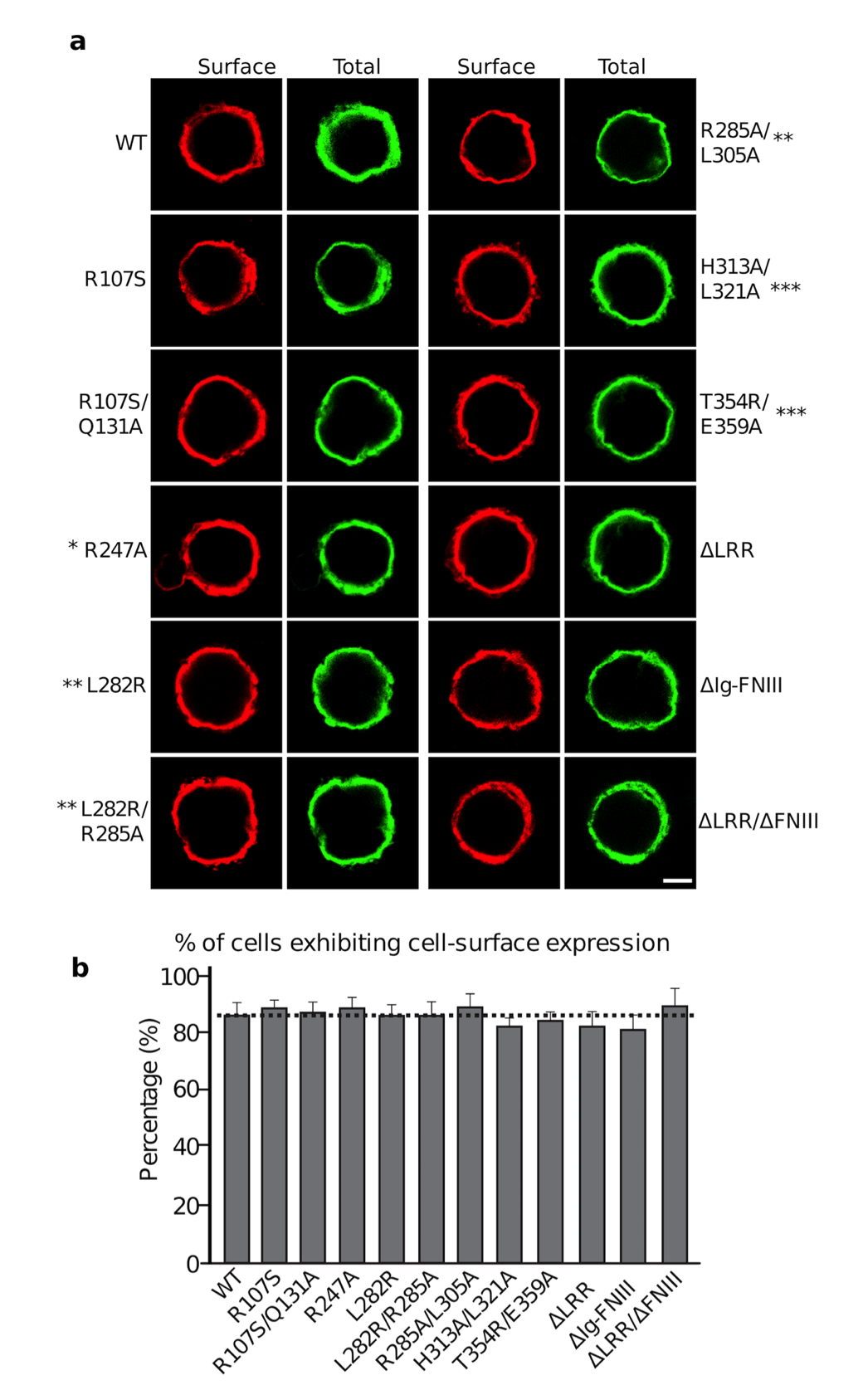
**

**Figure S10. Surface expression of SALM3 variants used in the current study.** A) Surface expression analysis of HEK293T cells expressing HA-tagged SALM3 WT, its indicated point mutants or deletion variants. Transfected cells were immunostained with mouse anti-HA antibodies and detected with Cy3-conjugated anti-mouse secondary antibodies under non-permeabilizing conditions, indicated as “Surface”, followed by permeabilization of cells. Cells were then immunostained with rabbit anti-HA antibodies and with FITC-conjugated anti-rabbit secondary antibodies, indicated as “Total”. This reflects expression both on the surface and intracellularly. Scale bar: 10 μm (applies to all images). B) Quantification of the proportion of cells exhibiting surface expression of SALM3. Data are means ± SEMs. ‘n’ denotes the number of cells, as follows: WT, n = 131; R107S, n = 121; R107S/Q131A, n = 151; R247A, n = 144; L282R, n = 129; L282R/R285A, n = 120; R285A/L305A, n = 109; H313A/L321A, n = 138; T354R/E359A, n = 134; ΔLRR, n = 132; ΔIg-FNIII, n = 128; and ΔLRR/ΔFNIII, n = 120.

**Table S1.** Amino acid residues present in SALM3 LRR dimer interface and the corresponding residues in SALM protein sequences. Interface residues in SALM3 structue were identified using the PISA server (<https://www.ebi.ac.uk/pdbe/pisa/>) with buried area percentage ≥ 75%. Multiple sequence alignment of human SALM proteins was calculated with MAFFT.

|  | **SALM5** | **SALM4** | **SALM3** | **SALM2** | **SALM1** |
| --- | --- | --- | --- | --- | --- |
| **Dimer interface** | Leu43 | Leu51 | Leu40 | Leu57 | Leu44 |
|  | Phe62 | Phe70 | Phe59 | Phe76 | Phe63 |
|  | Arg110 | Arg118 | Arg107 | Arg124 | Arg111 |
|  | Gln134 | Gln142 | Gln131 | Gln148 | Gln135 |
|  | Asn158 | Asn167 | Asn156 | Asn173 | Asn160 |
|  | Ser204 | Ser213 | Ser202 | Ser219 | Ser206 |
|  | Gly239 | Gly248 | Gly233 | Gly251 | Gly241 |
|  | Leu260 | Leu269 | Leu254 | Leu272 | Leu262 |
|  | Thr262 | Ala271 | Thr256 | Thr274 | Thr264 |

**Table S2.** Amino acid residues at SALM3-RPTPσ interface sites and the corresponding residues in the SALM5-RPTPδ complex. Interface residues were identified using PISA server (<https://www.ebi.ac.uk/pdbe/pisa/>) with buried surface area of ≥ 75%. Interactions in SALM3-RPTPσ interface are indicated according to type of interaction: hydrophobic (black line), hydrogen bonds (red line) or salt bridges (blue line). Differences are marked in bold for SALM5 residues. RPTP residues are all fully conserved (see text for closer description of interactions).

| **Interaction sites** | **SALM** | |  | **RPTPs** | |  |
| --- | --- | --- | --- | --- | --- | --- |
|  | **SALM5** | **SALM3** |  | **RPTPσ** | **RPTPδ** |  |
| Site I | Pro212  Cys246  Leu249  Trp250  Arg253 | Pro210  Cys240  Leu243  Trp244  Arg247 |  | Leu143  Val145  Tyr224  Gln202  Val225 | Leu134  Val136  Tyr215  Gln193  Val216 | Hydrophobic  Hydrophobic  Hydrophobic  Hydrogen bond  Hydrogen bond |
| Site II | Leu288  Ala310  Arg291  Arg311 | Leu282  Ala304  Arg285  Leu305 |  | Met305  Glu279  Asp275  Thr277  Met282  Tyr266 | Met296  Glu270  Asp266  Thr268  Met273  Tyr257 | Hydrophobic  Hydrogen bond  Salt bridge  Hydrogen bond  Hydrophobic  Hydrophobic |
| Site III | His319  Leu327  Ser360  Glu365 | His313  Leu321  Thr354  Glu359 |  | Met139  Asp138  Ala157  Leu155  Thr153  Lys144 | Met130  Asp129  Ala148  Leu146  Thr144  Lys135 | Hydrophobic  Hydrophobic  Hydrophobic  Hydrophobic  Hydrogen bond  Salt bridge |

**Table S3.** SAXS data collection and structural parameters.

|  | **SALM3 LRR-Ig -RPTP**σ | |
| --- | --- | --- |
| **Data collection parameters** | | |
| Beamline  Detector  Beam geometry  Wavelength (Å)  *q* range (Å^-1^) *  Exposure time (sec)/frame  Temperature (K) | B21, DLS  PILATUS 1M  0.2 x 0.2 mm  1.005  0.004-0.4  2.4  298K | |
| Concentration  Column | SALM3 (60 µM) and RPTPσ (80 µM) in 50 µl  Superdex 200 PC 3.2/30 | |
| **Structural parameters** | | |
| *I(0)* (relative) (from *P(r)*)  *R*_g_ (Å) (from *P(r)*)  *I(0)* (from Guinier)  *R*_g_ (Å) (from Guinier)  *D*_max_ (Å)  Porod volume *V*_p_ (Å^3^)  Excluded volume *V*_ex_ (Å^3^)  (*DAMMIN*/P1) | 0.20± 0.01  47.32± 0.01  0.20 ±0.01  46.41±0.1  171.00 ± 10  335000 ± 3000  400000 ± 4000 | |
| **Molecular mass determination (Da)** | | |
| From Porod volume (*V*_p_/1.7)  From SAXS MoW2 (Da)  From Guinier / *I(0)*  From sequence: protein / protein with glycans* (Da) | 197100 ± 1700  210000 ± 2100  168000  149444 / 163400 | |
| **Rigid body Modelling (*CORAL*)** | | |
| Symmetry  χ^2^ value | P1  1.5 | P2  2.5 |
| **Modelling parameters** | | |
| Shape reconstruction  Symmetry  NSD (var) / # of models  χ^2^ value | DAMMIN  P1  0.658 (0.016) /10  1.327 – 1.344 | |
| **Software employed** | | |
| Primary data reduction  Data processing  Computation of component model intensities  Model representations | *SCÅTTER*  *DATASW, PRIMUS*  *CRYSOL*  *PYMOL* | |

* assuming paucimannose-type glycans typical for insect cells

**Table S4**. Oligonucleotide pairs used for cloning and mutation of the gene constructs. On the first line the forward primer, on the second line the reverse primer.

| Plasmid constructs | Plasmid vector | Oligo pairs |
| --- | --- | --- |
| SALM3 LRR_17-284_ | pRMHA3 | 5’-TTTTGAATTCTGCCCGCTACCCTGTGTGTG-3’  5’-TTTTGGTACCGGCAATCAGCGGAGGCTCAC-3’ |
| SALM3 LRR-Ig-Fn _17-510_ | pDisplay | 5’-ATATATAGATCTTGCCCGCTACCCTGTGTG-3’  5’-ATATATGTCGACGGCTGGTAGCGTAGAGAAGTGG-3’ |
| SALM3 LRR _17-284_ | pDisplay | 5’-ATATATAGATCTTGCCCGCTACCCTGTGTG-3’  5’-ATATATGTCGACAGGCTCACAGGAGAACTCTCCCTC-3’ |
| SALM3 Ig_280-367_ | pDisplay | 5’-ATATATAGATCTGAGCCTCCGCTGATTGCC-3’  5’-ATATATGTCGACCCGGAGCTCCACTCGGG-3’ |
| SALM3 Ig-Fn_280-502_ | pDisplay | 5’-ATATATAGATCTGAGCCTCCGCTGATTGCC-3’  5’- ATATATGTCGACGGCTGGTAGCGTAGAGAAGTGG-3’ |
| SALM3 Q131A | pDisplay | 5’-CTCAGTGGCAATGCACTGGGCCGCATCGC-3’  5’-GCGATGCGGCCCAGTGCATTGCCACTGAG-3’ |
| SALM3 R247A | pDisplay | 5’-CTGCTGTGGCTGCGGGCGCTGGCCCGGC-3’  5’-GCCGGGCCAGCCGCGCCAGCCACAGCAG-3’ |
| SALM3 L282R | pDisplay | 5’-CTGTGAGCCTCCGCGGATTGCCCGGCACAC-3’  5’-GTGTGCCGGGCAATCCGCGGAGGCTCACAG-3’ |
| SALM3 R285A | pDisplay | 5’-CTCCGCTGATTGCCGCGCACACACAGCGCCTG-3’  5’-CAGGCGCTGTGTGTGCGCGGCAATCAGCGGAG-3’ |
| SALM3 R303A/L305A | pDisplay | 5’-CACCCTACGGTGCGCGGCCGCTGGTGACCCTGTACC-3’  5’-GGTACAGGGTCACCAGCGGCCGCGCACCGTAGGGTG-3’ |
| SALM3 H313A | pDisplay | 5’-GTACCTACCATGGCCTGGGTTGGCCCTG-3’  5’-CAGGGCCAACCCAGGCCATGGTAGGTAC-3’ |
| SALM3 L321A | pDisplay | 5’-CCTGATGACAGGGCGGTTGGCAACTCTTC-3’  5’-GAAGAGTTGCCAACCGCCCTGTCATCAGG-3’ |
| SALM3 T354R | pDisplay | 5’-CCTGCATTGCCCGCAACCCTGCTGGTG-3’  5’-CACCAGCAGGGTTGCGGGCAATGCAGG-3’ |
| SALM3 E359A | pDisplay | 5’-CAACCCTGCTGGTGCGGCCACAGCCCGAGTG-3’  5’-CACTCGGGCTGTGGCCGCACCAGCAGGGTTG-3’ |
